# The *Chlamydia trachomatis* PmpD adhesin forms higher order structures through disulphide-mediated covalent interactions

**DOI:** 10.1371/journal.pone.0198662

**Published:** 2018-06-18

**Authors:** Wayne Paes, Adam Dowle, Jamie Coldwell, Andrew Leech, Tim Ganderton, Andrzej Brzozowski

**Affiliations:** 1 York Structural Biology Laboratory, University of York, York, United Kingdom; 2 Centre for Immunology and Infection, University of York, York, United Kingdom; 3 Technology Facility, University of York, York, United Kingdom; Centre National de la Recherche Scientifique, Aix-Marseille Université, FRANCE

## Abstract

*Chlamydia trachomatis* (*Ct*) is the most common sexually transmitted bacterial pathogen, and the leading cause of infectious blindness worldwide. We have recently shown that immunization with the highly conserved antigenic passenger domain of recombinant *Ct* polymorphic membrane protein D (rPmpD) is protective in the mouse model of *Ct* genital tract infection, and previously, that ocular anti-rPmpD antibodies are elicited following vaccination. However, the mechanisms governing the assembly and structure-function relationship of PmpD are unknown. Here, we provide a biophysical analysis of this immunogenic 65 kDa passenger domain fragment of PmpD. Using differential cysteine labeling coupled with LC-MS/MS analysis, we show that widespread intra- and intermolecular disulphide interactions play important roles in the preservation of native monomeric secondary structure and the formation of higher-order oligomers. While it has been proposed that FxxN and GGA(I, L,V) repeat motifs in the Pmp21 ortholog in *Chlamydia pneumoniae* mediate self-interaction, no such role has previously been identified for cysteine residues in chlamydial Pmps. Further characterisation reveals that oligomeric proteoforms and rPmpD monomers adopt β–sheet folds, consistent with previously described Gram-negative bacterial type V secretion systems (T5SSs). We also highlight adhesin-like properties of rPmpD, showing that both soluble rPmpD and anti-rPmpD serum from immunized mice abrogate binding of rPmpD-coated beads to mammalian cells in a dose-dependent fashion. Hence, our study provides further evidence that chlamydial Pmps may function as adhesins, while elucidating yet another important mechanism of self-association of bacterial T5SS virulence factors that may be unique to the *Chlamydiaceae*.

## Introduction

*Chlamydia trachomatis* (*Ct*) is a Gram-negative obligate intracellular pathogen of humans, and the etiological agent of blinding trachoma and sexually transmitted diseases [1]. Ocular *Ct* serovars (A-C) primarily infect human conjunctival epithelial cells, with repeated infections leading to corneal opacity and subsequent blindness. Collectively, serovars (D-K) and lymphogranuloma venereum (LGV) strains (L1-L3) are the most common sexually transmitted bacterial pathogens worldwide, responsible for ~131 million new cases each year [2]. Urogenital infections are asymptomatic in 70–90% of women, with untreated ascending infections the main cause of substantial morbidity and post-infection sequelae such as pelvic inflammatory disease, ectopic pregnancies and infertility [3]. Thus, efficacious vaccine design remains a high public health priority.

In light of recently identified chimeric variation and recombination within the *ompA* genetic locus encoding the major outer membrane protein (MOMP) which comprised the mainstay of chlamydial vaccine development for over two decades [4], chlamydial *p*olymorphic *m*embrane *p*roteins (Pmps) have more recently elicited widespread interest as candidate vaccine immunogens. This is largely due to their surface-exposed nature and accessibility to neutralising antibodies, a putative role in mediating adhesin-like function, as well as the abundance of T-cell epitopes on these highly immunogenic proteins that are able to prime robust cell-mediated immunity [5–8]. Pmps are part of a large and diverse family of type V autotransporter (T5SSs) proteins within the *Chlamydiaceae*. T5SSs are thought to comprise the largest family of secreted proteins in Gram-negative bacteria [9], and *Ct* possesses nine Pmps (Pmp A-I) within its genomic repertoire, representing roughly 13% of the total genomic coding capacity [10]. The maintenance of such a large family of Pmps in a bacterial phylum that has undergone evolution by genomic reduction, suggests either crucial or redundant roles for these proteins in the life cycle of *Ct*.

Of these Pmps, PmpD is the most highly conserved, with 99.15% sequence identity between the 18 reference serovars [11]. Swanson *et al*. report membrane-associated and soluble forms of PmpD, with flower-like oligomeric structures (~23nm) observed on infectious elementary bodies (EBs), that are thought to comprise full length (~155 kDa) and processed (~73 kDa and ~82 kDa) forms of PmpD, while Kiselev *et al*. reported processing of the 157 kDa passenger domain to 65 kDa and 80 kDa fragments in intracellular metabolically active reticulate body (RB) forms [12, 13]. Furthermore, post-translational processing of PmpD orthologs has been reported in *Chlamydia pneumoniae* and *Chlamydophila abortus* [14, 15]. These studies demonstrate that PmpD and its orthologs are surface-exposed and undergo post-translational processing in a range of pathogenic chlamydial species.

Concomitant with nascent advances in the genetic manipulation of *Ct*, investigation of PmpD function *in vivo* was addressed following truncation of the protein by chemical mutagenesis using a targeted reverse genetics approach [16]. The *pmpD* mutant showed aberrant inclusion morphology and reduced association of RBs with the inclusion membrane, but no obvious growth defects. In addition, the mutant was significantly attenuated for virulence in human endocervical and conjunctival cells when compared to the wild-type strain, suggesting that PmpD plays a role in early interactions with host cells, as well as during biphasic development within the inclusion membrane.

Leading on from these observations, we have recently shown that immunization with the N-terminal 65 kDa passenger domain of rPmpD elicits protection against *Ct* genital tract infection, which correlates with high levels of rPmpD-specific serum and cervico-vaginal IgG titres even in the absence of a significantly elevated rPmpD-specific Th1-type cellular response [8, 17]. Previously, we found that intramuscular immunization with rPmpD elicited ocular antibodies, suggesting the potential utility of this pan-conserved antigen in clinical vaccine development against both sexually transmitted infections and trachoma [8, 17].

An accumulating body of evidence in *Chlamydia pneumoniae* is now beginning to inform the molecular interactions between Pmp21 (the ortholog of PmpD) and mammalian cells, and although no host cell ligand has been defined for any *Ct* Pmp, it has been shown that these outer membrane proteins function as adhesins. It was first observed that FxxN and GGA(I,L,V) repeat motifs influence host cell adhesion and self-interaction, and subsequently, that mutating one of these repeat motifs in a truncated 23 kDa C-terminal recombinant Pmp21 fragment results in reduced ability to form oligomers [5, 18, 19]. PmpD contains 17 GGA(I,L,V) and 19 FxxN tetrapeptide sequences that may indeed play similar roles in *Ct*, as these repeat motifs are a characteristic feature of chlamydial autotransporter passenger domains, and markedly over-represented in Pmps relative to the rest of the proteome [20]. This suggests that chlamydial membrane protein assembly and host cell interactions may occur through unique interfacial contacts particular to this ancient bacterial phylum. Indeed, in addition to repeat motifs, the chlamydial outer membrane is also unique when compared to all other known Gram-negative phyla due to the presence of an extensively oxidized disulphide-linked outer membrane complex, which undergoes morphological changes during the life cycle of *Ct*. *Ct* EBs consist primarily of MOMP and the two major cysteine-rich proteins OmcA and OmcB [21]. However, following internalisation, the EB membrane undergoes disulphide reduction and expansion within the intracellular chlamydial inclusion as the bacterium differentiates into larger metabolically active RBs [21].

In this study, we investigated the mechanism of assembly and adhesion properties of the recombinant N-terminal 65 kDa passenger domain fragment of PmpD (rPmpD) *in vitro*. We provide a biophysical characterization of rPmpD, identifying for the first time, a unique role for disulphide bonds in oligomerization and self-interaction of this T5SS protein, as well as in the preservation of monomeric secondary structure, showing that all 18 cysteine residues are disulphide-bonded in the dominant proteoforms. Circular dichroism analysis shows that rPmpD consists primarily of β–sheet secondary structure, which is consistent with biophysical analyses presented for the truncated 23 kDa Pmp21 domain in *Chlamydia pneumoniae*, as well as experimentally solved crystal structures of other Gram-negative bacterial T5SSs [18, 22]. Finally, we show that rPmpD possesses adhesin-like characteristics, and that both soluble rPmpD and anti-rPmpD serum from immunized mice abrogate adhesion of rPmpD-coated beads to the mammalian Hak cell line, highlighting a putative role for neutralizing anti-rPmpD antibodies in *in vitro* studies, which is consistent with our previous observations *in vivo* [8].

## Materials and methods

### Purification and preparation of rPmpD

Bacteria were induced with IPTG at an optical density of between 0.6–1.0. Following overnight expression at 37°C, bacteria were lysed by sonication in the following lysate buffer (20mM Tris-HCl, pH 8.0 50mM NaCl, 5mM EDTA, 0.5% Triton-X100 and 0.1mM phenylmethylsulfonyl fluoride (PMSF)). Cells from 4 litres of culture were lysed in 250 mL of lysate buffer and sonicated for a total of 10–15 minutes on ice in 10s bursts with 10s intervals. Following sonication, 10mM MgSO4 was added to chelate EDTA, and lysozyme (0.1 mg/mL) subsequently added to the lysate and incubated at RT for 20min to facilitate cell lysis. Cell lysate was centrifuged (6000 rpm for 15 minutes) to collect inclusion bodies, and the pellet resuspended completely once more by sonication in the lysate buffer. The final wash step was conducted using lysate buffer without Triton-X100: 20mM Tris-HCl, pH 8.0 50mM NaCl, 5mM EDTA.

The washed inclusion body pellets were dispersed completely by sonication and then dissolved overnight at 4°C by stirring vigorously, in 20mM Tris buffer (pH 8.0) containing 6M GdHCl. 100 mL for 2L of culture was used at this dissolution stage. The following day, the solution was centrifuged (6000 rpm for 20 minutes) to remove any insoluble particulates, and refolding of the rPmpD was conducted using step-wise dialysis by altering the concentration of GdHCl from 6M to 4M to 2M to 1M over the course of 3 days at 4°C. For refolding under reducing conditions, 5mM reduced glutathione and 0.5mM oxidized glutathione were added to the refolding buffer.

Recombinant PmpD was purified using a nickel-affinity chromatography column (GE Healthcare) pre-equilibrated with binding buffer (20mM TrisHCl pH 8.0, 50mM NaCl, 10mM imidazole), and protein was eluted with a linear imidazole gradient (10–500mM). Eluted fractions were analysed by SDS–PAGE and those containing overexpressed protein (approximately 90% pure) were pooled, concentrated and then applied onto an S200 16/60 Superdex gel-filtration column (GE Healthcare) pre-equilibrated with 20mM TrisHCl pH 8.0, 50mM NaCl. Eluted fractions containing rPmpD were flash frozen in liquid nitrogen and stored at −80°C until further use.

Prior to use in *in vitro* assays and *in vivo* experiments, LPS phase separation was performed as previously described [23]. Briefly, Triton X-114 detergent was added to rPmpD oligomeric or monomeric fractions to a final concentration of 1% (v/v) and incubated at 4°C for 30 mins with constant rotation. The mixture was then transferred to a water bath at 37°C for 10mins followed by ultracentrifugation (20,000 g, 10 mins) to separate LPS-containing micelles from the aqueous fraction. The aqueous supernatant was collected and endotoxin concentration measured using the Limulus Amebocyte Lysate assay. The LAL assay was utilised to determine the amount of endotoxin in the rPmpD preparation, which was found to be below 0.1 EU/mg.

### Dynamic light scattering

The polydispersity of rPmpD oligomeric and monomeric fractions was analysed using dynamic light scattering (DLS). Measurements were carried out at 20°C with the protein at 1 mg/mL in size exclusion buffer using the DynaPro Dynamic Light Scattering system (Protein Solutions).

### Circular dichroism spectroscopy

Circular-dichroism (CD) spectra were recorded at 20°C with a Jasco J-180 CD spectrophotometer using a 0.1 cm path-length quartz cell. Experiments were carried out in 20mM Tris/HCl buffer pH 8.0. The protein concentrations in the samples were 0.2 mg/mL. Random error and noise were reduced for each spectrum by averaging three scans in the wavelength range 260nm–195 nm. The signal acquired for the buffer used for dilution of the proteins was subtracted from the spectra acquired for the proteins.

### SDS- and Native PAGE analyses

SDS-PAGE rPmpD samples were either denatured for 5 mins at 95°C in sample loading buffer (0.5M Tris-HCl–pH 6.8, 10% (w/v) glycerol, 2% (w/v) SDS, 0.005% Bromophenol Blue) prior to gel loading, or exposed to loading buffer containing either β–mercaptoethanol and/or SDS without heating (as explicitly stated). Unstained broad range (2–212 kDa) protein marker (NEB) was added to determine protein molecular weight, and samples were separated at 200V for 35 mins in running buffer composed of 25 mM Tris, 250 mM glycine, 0.1% (w/v) SDS, pH 8.4, following loading on to 12% acrylamide SDS gels. All running and loading buffers for Native PAGE were composed of the same constituents as in SDS PAGE, but without the addition of SDS detergent. Native samples were separated at 100V for 1hr on gels composed of 5% acrylamide (without SDS). rPmpD concentration was measured using the Pierce Coomassie (Bradford) Protein assay kit (ThermoFisher), with absorbance measured at 595nm on a UV Eppendorf Biophotometer after blanking with elution buffer. Unknown protein concentrations were determined from the BSA protein standard curve provided.

### Tryptic digests

Limited proteolysis was carried out on the 65 kDa rPmpD passenger domain in 10 μl reaction mixtures with a final protein concentration of 0.2 mg mL^−1^ in 50 mM Tris–HCl (pH 7.5) and 50–150 mM NaCl. Trypsin (Hampton Research) was added, and the mixture incubated between 0.5–30 mins. Following quenching of the reaction by addition of 1–10mM PMSF, the products were analysed by SDS–PAGE.

### Differential cysteine labelling

A 50μL aliquot of rPmpD in aqueous 20mM Tris pH 8.0 and 50mM NaCl, was diluted to 100 μL with aqueous 80 μM iodoacetamide and incubated for 30mins at room temperature. The sample was buffer exchanged into aqueous 50mM ammonium bicarbonate using an Amicon 30 kDa spin filter (5 mins, 14,000 g). Concentrated protein was taken up to a final volume of 120μL with aqueous 50 mM ammonium bicarbonate and split into three 40 μL aliquots for parallel digestion with trypsin (Promega), chymotrypsin (Roche) and Asp-N (Sigma) sequencing grade proteases. A 0.2 μg aliquot of protease in 10 μL of 50mM ammonium bicarbonate was added to each and incubated at 37°C for 16h. Each digest was split into two aliquots, one reduced with the addition of 2 μL 50 mM tris (2-carboxyethyl) phosphine (TCEP) and the other treated with 2 μL 50 mM ammonium bicarbonate as a control. Both the aliquot containing the reductant and the control were heated at 60°C for 1h before alkylating with 1.25 μL of 0.2 M methyl methanethiosulfonate (MMTS) and incubation at room temperature for 30 mins. Digests were acidified with aqueous 0.1% trifluoroacetic acid and 10 μL aliquots taken from each of the three protease-derived peptide mixtures and combined to give one reduced and one non-reduced sample, each of which had been subjected to the same alkylation regime.

Samples were loaded onto an UltiMate 3000 RSLCnano HPLC system (Thermo) equipped with a PepMap 100 Å C_18_, 5μm trap column (300 μm x 5 mm Thermo) and a PepMap, 2 μm, 100 Å, C_18_ EasyNano nanocapillary column (75μm x 150mm, Thermo). The trap wash solvent was aqueous 0.05% (v:v) trifluoroacetic acid and the trapping flow rate was 15μL/min. Separation was achieved by linear multi-step gradient elution of two solvents: solvent A, aqueous 1% (v:v) formic acid, and solvent B, aqueous 80% (v:v) acetonitrile containing 1% (v:v) formic acid, with a capillary column flow rate of 300 nL/min and column temperature of 40°C. The linear multi-step gradient profile was: 3–10% B over 7 mins, 10–35% B over 30 mins, 35–99% B over 5 mins and then proceeded to wash with 99% solvent B for 4min.

The nanoLC system was interfaced with an Orbitrap Fusion hybrid mass spectrometer (Thermo) with an EasyNano ionisation source (Thermo). Positive ESI-MS and MS^2^ spectra were acquired using Xcalibur software (version 4.0, Thermo). Instrument source settings used: ion spray voltage, 1,900 V; sweep gas, 0 Arb; ion transfer tube temperature; 275°C. MS^1^ spectra were acquired in the Orbitrap with: 120,000 resolution, scan range: *m/z* 375–1,500; AGC target, 4e^5^; max fill time, 100 ms. Data dependant acquisition was performed in top speed mode using a fixed 1s cycle, selecting the most intense precursors with charge states >1. Easy-IC was used for internal calibration. Dynamic exclusion was performed for 50s post precursor selection and a minimum threshold for fragmentation was set at 5e^3^. MS^2^ spectra were acquired in the linear ion trap with: scan rate, turbo; quadrupole isolation, 1.6 *m/z*; activation type, HCD; activation energy: 32%; AGC target, 5e^3^; first mass, 110 *m/z*; max fill time, 100ms. Acquisitions were arranged by Xcalibur to inject ions for all available parallelizable time.

### LC-MS/MS data analysis

Thermo .raw files were imported onto PEAKS studio 8.0 for peak picking and peptide identification. Database searching was performed against an in-house FASTA file containing the expected sequence of His_6_-tagged rPmpD, with search criteria specified as: Enzyme, none; Variable modifications, oxidation (M), carbamidomethylation (C), beta-methylthiolation (C); Peptide tolerance, 3ppm; MS/MS tolerance, 0.5 Da; Instrument, ESI-TRAP; Fragmentation, HCD. PEAKS-DB results were filtered to achieve a 1% peptide false discovery rate.

Peak lists in .raw format were imported into Progenesis QI (nonlinear–Ver. 3) for relative peak area-based quantification. LC-MS runs aligned with the reduced sample chosen as the reference. A combined peak list was exported in .mgf format for database searching. Mascot Daemon (version 2.5.1, Matrix Science) was used to submit the search to the Mascot program (Matrix Science Ltd., version 2.5.1). Search criteria were identical to the PEAKS search with the exception that an independent search was carried out for each protease specificity. Search results were passed through Mascot Percolator to achieve a 1% peptide false discovery rate and filtered to require a minimum expected score of 0.05 for individual matches. The Mascot .XML result files were imported into Progenesis QI and peptide identifications associated with precursor peak areas. MS^1^ peak areas were exported without normalisation for all identified PmpD peptides. Peak areas for carbamidomethylated and methylthiolated variants of the same peptide sequence and charge state were aligned and expressed as the relative percentage of total peptide from intensity across the reduced and non-reduced sample.

### PmpD conjugation to carboxylate-modified beads

Covalent coupling of oligomeric rPmpD or bovine serum albumin (BSA) (Sigma Aldrich) to carboxylate-modified L4530 fluorescent beads (Sigma Aldrich) was performed by reaction with 1mM *N*-Ethyl-*N*′-(3-dimethylaminopropyl) carbodiimide hydrochloride (EDAC) (Sigma Aldrich) in 50mM MES pH 6.1. 50 μg/mL, 25 μg/mL, 2.5 μg/mL and 0.25 μg/mL concentrations of rPmpD (or 200 μg/mL BSA) were used for coating beads. The mixture was incubated at 20°C with gentle shaking for 1h and the reaction was stopped by centrifugation of the suspension, discarding the supernatant, and washing the pellet twice in binding buffer (RPMI containing 10% BSA (w/v), 10mM HEPES, pH 7.5) to remove all traces of EDAC. Coupling of rPmpD protein to the beads was verified on SDS-PAGE by inspection of the reaction supernatant and boiling of coated beads at 95°C for 5mins.

### Hak cell bead adhesion and inhibition assays

Serial dilutions of beads (1:20, 1:50 and 1:100) were added to Hak cells (ATCC) in binding buffer to determine the optimal dilution for subsequent assays. Adhesion assays with rPmpD-coated or control (naked or BSA-coated) beads were performed on Hak cell monolayers (70–80% confluent) in 96-well plates. For all assays, 1 mL of 1% L4530 bead suspension was used, and beads were incubated for 1 h at 4°C to initiate binding. Unattached beads were removed by twice washing with PBS, and cells were fixed with methanol. Binding was quantified following Giemsa staining, by counting the number of attached beads per 50 cells in triplicate fields of view under phase microscopy. Filtered Giemsa stain solution consisted of 3.6 mL Sorensen A solution (9.5g/l NaHPO_4_ (Sigma)), 1.4 mL Sorensen B solution (9.07g/l KH_2_PO_4_ (Sigma), 45 mL de-ionized water and 5.55 mL Giemsa stain (VWR International).

In competitive inhibition assays, Hak cell monolayers were pre-incubated with four dilutions (50 μg/mL, 25 μg/mL, 2.5 μg/mL and 0.25 μg/mL) of soluble rPmpD in binding buffer in 96-well plates for 1hr at 4°C to allow for binding. Following two washes in PBS to remove unbound protein, a 1:50 dilution of a 1% solution of L4530 fluorescent beads were added and incubated for 2h at 4°C to allow for binding.

For the serum neutralisation assays, a 1:50 dilution of a 1% solution of rPmpD-coated L4530 fluorescent beads were separately pre-incubated with two dilutions (1:50, 1:100) of heat-inactivated pre-immune or anti-rPmpD immune serum (obtained from immunized C57BL/6 mice) for 1h at 37°C with gentle shaking to enable binding of antibodies to the beads. Bead-serum mixtures were then added to Hak cell monolayers (as described above), in 96-well plates, and incubated for 2h at 4°C to allow for binding. In both assays, unattached beads were removed by twice washing with PBS, and cells were fixed with methanol. Enumeration of attached beads was implemented as described above.

### Mouse immunizations

All animal care and experimental procedures were performed under UK Home Office License (Ref # PPL 70/7805) and with approval from the Animal Procedures and Ethics Committee of the University of York. 6–8 week old female C57BL/6 mice (n = 5) were bred and maintained in individually vented cages under specific pathogen-free conditions at the University of York, and monitored daily for any signs of distress. Animal weight was recorded weekly to monitor any signs of weight loss. Mice were immunized subcutaneously with three doses of monomeric rPmpD in combination with an oil-in-water emulsion formulation of a second-generation lipid adjuvant (SLA-SE) as previously described [8]. To obtain serum for neutralization assays, mice were euthanized 55 days post initial immunization using a rising concentration of carbon dioxide, and 0.5–1 mL of blood was collected via cardiac puncture using a 25G needle. Pre-immune and anti-rPmpD serum was heat-inactivated prior to use in neutralization assays by heating at 56°C for 30 mins.

## Results

### Recombinant PmpD passenger domain purifies as a complex array of homo-oligomers with monomeric species in low abundance

**Fig 1:**
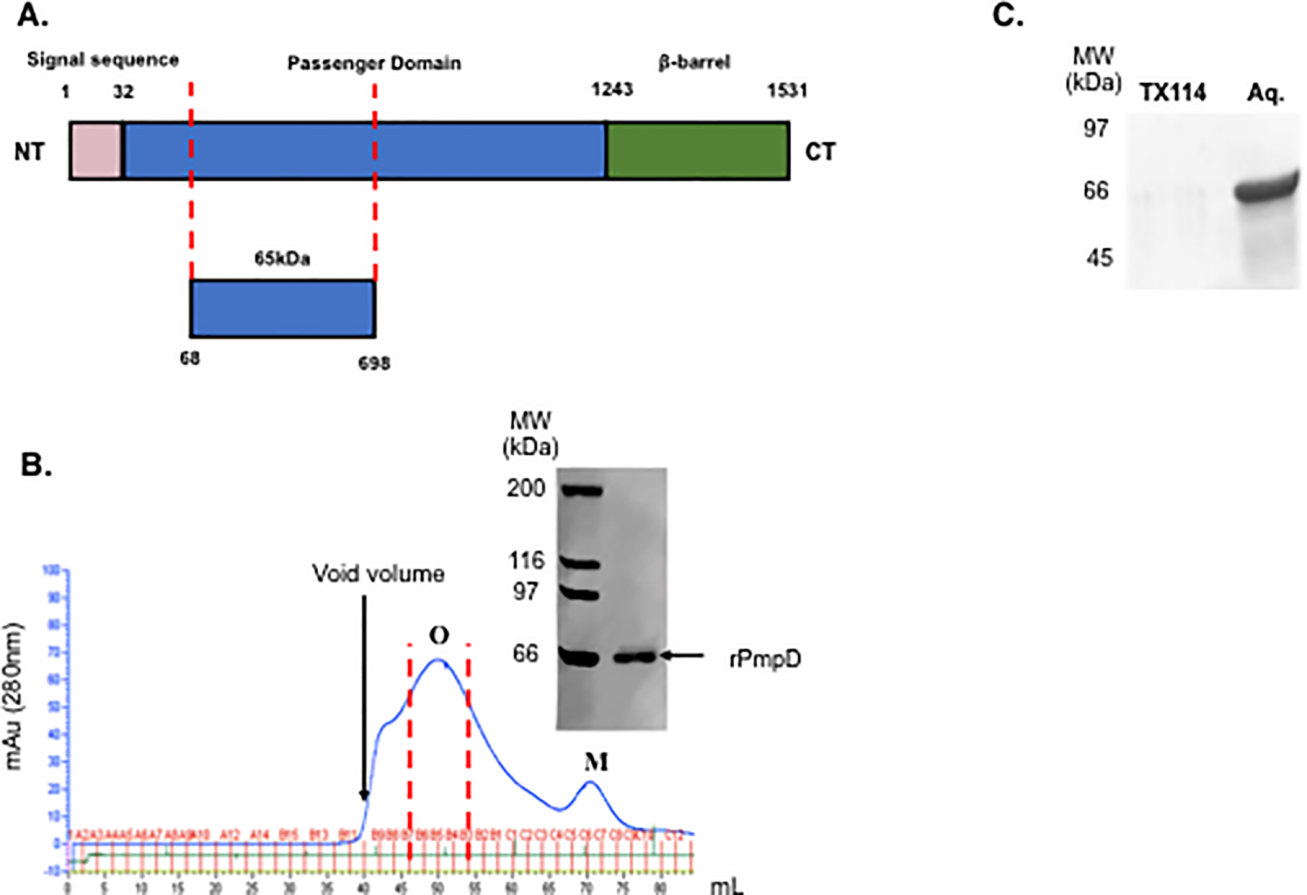
*Chlamydia trachomatis* polymorphic membrane protein D (PmpD) schematic and elution profile. (**A**) *Ct* PmpD displays the archetypal characteristic three-domain structure of Gram-negative bacterial type V autotransporters. Signal sequence (pink), passenger domain (blue) and β-barrel autotransporter (green) are depicted with the corresponding amino acid positions. The 65 kDa passenger domain fragment used in this study is depicted below, as detected by Kiselev *et al*. in infected cells [13]. (**B**) Soluble 65 kDa rPmpD elutes as a mixture of oligomeric species (‘O’) with substantially lower concentrations of soluble monomeric protein (‘M’). SDS-PAGE shows the chemical purity of the eluate (inset), and is representative of each collected fraction. Fractions B7-B3 (within dashed red lines) were pooled, and comprise the ‘oligomeric’ fraction used in all downstream experiments. The flow rate used was 0.3 ml/min, and the elution profile is representative of independent replicate purifications (n = 5). (**C**) rPmpD partitions exclusively in to the aqueous fraction following phase separation, indicative of predominantly hydrophilic properties of the passenger domain.

The 65 kDa PmpD fragment identified by Kiselev *et al*. [13] using mass spectrometry analysis (**Fig 1A**) was over-expressed in *E*.*coli* strain BL21(DE3) and extracted under denaturing conditions (6M GdHCl). After step-wise dialysis, refolded protein was purified with a C-terminal His_6_-tag using immobilized metal affinity chromatography, followed by size exclusion chromatography (**Fig 1B**). rPmpD elutes as a complex mixture of oligomers with a broad peak (42–65 mL) emerging shortly after the 40 mL void volume of the S200 16/60 column, with a much smaller delineated monomeric peak between 67–75 mL. Reducing SDS-PAGE analysis of all fractions showed exclusive representation of the rPmpD 65 kDa domain (**Fig 1B, inset**), and the elution profile was consistent in multiple subsequent purifications (data not shown), indicating robust methodological reproducibility. Refolding in the presence of 5mM reduced glutathione and 0.5mM oxidized glutathione did not result in a change in the SEC elution profile and a shift to monomeric protein (not shown), likely suggesting that oligomers comprise the dominant native state of the protein *in vitro*. In contrast to the *Ct* MOMP that is highly hydrophobic and poorly soluble in the absence of detergents and lipid bilayers [24], both monomeric and oligomeric rPmpD were found to partition almost exclusively in to the aqueous rather than the detergent phase during Triton X-114 phase separation of LPS (**Fig 1C**), showing predominantly hydrophilic properties. Interestingly, the elution profile of rPmpD closely resembles that of the Pmp21 23 kDa domain previously described [18], and we next sought to determine the biophysical characteristics of the purified *Ct* rPmpD 65 kDa passenger domain fragment, as well as delineate the biochemical mechanisms of *Ct* PmpD interaction and native secondary structure.

### rPmpD oligomers demonstrate polydispersity and consist primarily of 𝛃–sheet secondary structure

**Fig 2:**
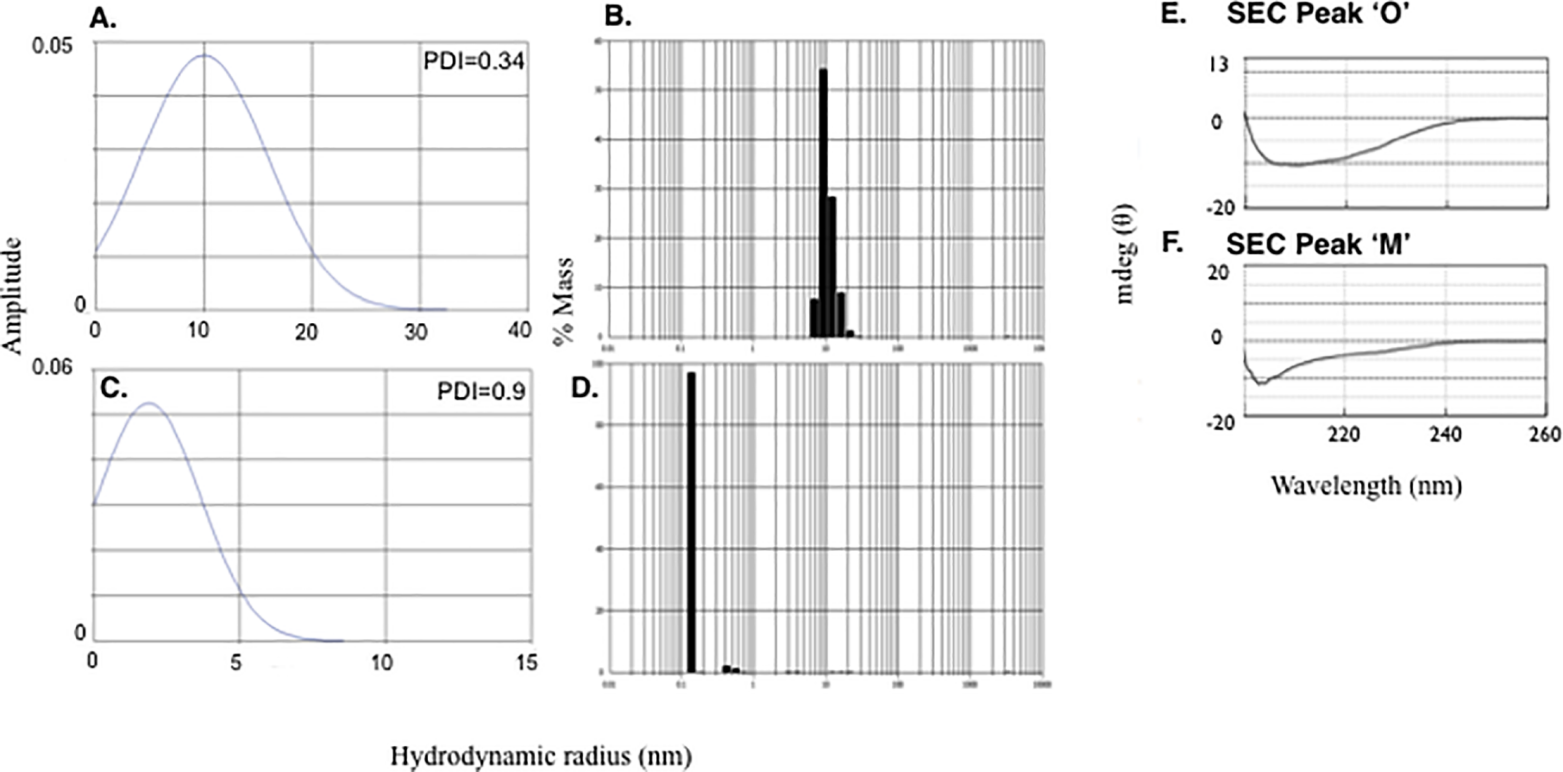
rPmpD monomeric and oligomeric forms show differing polydispersity and are comprised of β-sheet secondary structure. rPmpD fractions were analysed using dynamic light scattering and circular dichroism. The polydispersity index is displayed above the frequency histograms of (**A**) oligomeric and (**C**) monomeric fractions. Regularization histograms showing % mass versus hydrodynamic radius (nm) for (**B**) oligomeric and (**D**) monomeric rPmpD fractions are also displayed, indicating the absence of larger molecular weight proteoforms in monomeric rPmpD following size exclusion chromatography. Soluble oligomeric **(E)** and monomeric **(F)** fractions of rPmpD were analysed using circular dichroism. Spectra for oligomeric (Peak ‘O’) and monomeric (Peak ‘M’) rPmpD fractions are indicative of predominantly β-sheet secondary structure interspersed with random coil elements, similar to the Pmp21 ortholog in *C*.*pneumoniae* [18].

Dynamic light scattering (DLS) was carried out to assess the size distribution of species within the two fractions. Polydispersity index (PDI) was used as a measure of sample homogeneity, and defined as the ratio of the average molecular mass to the number average molecular mass, with a PDI = 1.0 indicating complete monodispersity. Histograms plotting hydrodynamic radius (nm) against particle size distribution indicate a PDI of 0.34 for the oligomeric fraction (**Fig 2A**), highlighting a complex mixture of higher order rPmpD species, while monomeric rPmpD has a PDI of 0.9, reflecting nearly complete monodispersity (**Fig 2C**). Regularization histograms showing the percentage (by mass) of particles of a given hydrodynamic radius within the samples are also depicted for oligomeric (**Fig 2B**) and monomeric (**Fig 2D**) rPmpD proteoforms. Oligomeric and monomeric forms of rPmpD were further characterized by using circular-dichroism (CD) spectroscopy. The molar ellipticity of rPmpD exhibits a shallow minimum at 215–220 nm, typically associated with the predominance of β-sheet structure (**Fig 2E and 2F**), and consistent with experimentally solved β-helical structures of Gram-negative T5SSs [25].

### Disulphide bonding mediates oligomerization and governs native secondary structure of rPmpD

**Fig 3:**
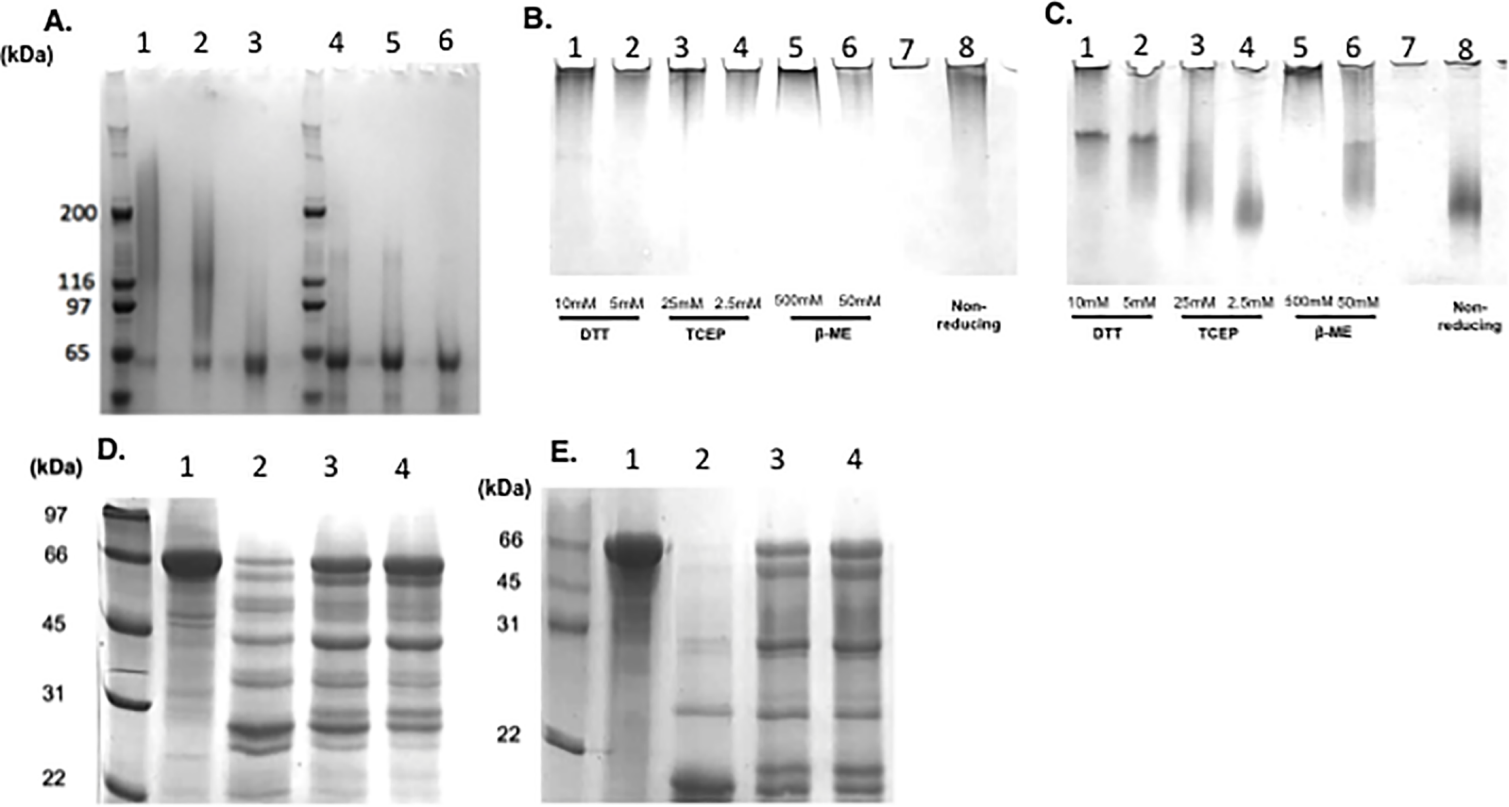
Inter- and intramolecular disulphide bonding critically influence the biophysical properties of rPmpD. (**A**) Oligomeric (Lanes 1–3) and monomeric (Lanes 4–6) rPmpD samples were incubated with loading buffer in the absence or presence of 0.5 M β-mercaptoethanol (β-ME). Native gels were also run with **(B)** oligomeric or **(C)** monomeric rPmpD samples incubated in the absence of SDS with differing concentrations of three reducing agents– 10 mM or 5 mM DTT (Lanes 1–2), 25 mM or 2.5 mM TCEP (Lanes 3–4) and 0.5M or 50 mM β-ME (Lanes 5–6). Non-reduced sample was run as a control (Lane 8). **(D)** oligomeric or **(E)** monomeric rPmpD were incubated in the presence of trypsin for 30 min, 5 mins, or 2 mins (Lanes 2–4, respectively). Undigested control samples were run in Lane 1 of each gel.

When rPmpD was separated on reducing and non-reducing SDS gels (**Fig 3A**), the monomer migrated alongside the 66 kDa molecular weight marker in both the absence and presence of reducing agent (Lanes 3 and 6). However, oligomeric rPmpD failed to dissociate completely in the absence of 0.5M β–ME in the loading buffer (Lanes 1 and 2), indicative of predominantly intermolecular disulphide bonding in the majority of species present within the mixture, with some non-covalent interactions also present in these proteoforms that do dissociate in to constitutive monomers. SDS, an anionic detergent, is sufficient to disrupt this proportion of non-covalent interactions within the rPmpD oligomers.

Native (SDS-free) gels of monomeric and oligomeric rPmpD showed migration of monomeric rPmpD occurs in the absence of β–ME (**Fig 3C,** Lane 8), whereas the oligomeric fraction failed to enter and migrate in to the 5% acrylamide gel, likely due to its high molecular weight (**Fig 3B,** Lane 8). The addition of reducing agents to oligomers was expected to initiate dissociation in to constituent monomeric subunits, however, contrary to our expectation, the use of three different reducing agents (β–ME, TCEP or DTT) at a range of concentrations had no effect on entry and migration of the oligomer in to native gels (**Fig 3B,** Lanes 1–6). Interestingly, the migration of rPmpD monomer was also affected by the presence of reducing agents, increasing concentrations of which led to reduced entry and mobility of the protein (**Fig 3C,** Lanes 1–6). These observations suggest that reduction of disulphide bonds in the monomer causes the formation of higher order aggregates by disrupting native protein structure in a concentration-dependent manner, which subsequently precludes protein entry in to the native gel. Thus, we hypothesized that in addition to mediating intermolecular interactions, disulphide bonding is essential for preservation of native PmpD structure. To further address the apparent complexity of this disulphide-bonded network, differential alkylation of monomeric rPmpD was undertaken in an attempt to identify reduced cysteines that could potentially form intermolecular disulphide bridges responsible for this oligomerisation.

### Oligomerisation protects against digestion with trypsin

Both rPmpD monomer and oligomers were subjected to tryptic digest as described in the Materials and Methods. Notable differences were observed between oligomeric (**Fig 3D**) and monomeric rPmpD proteoforms (**Fig 3E**). The 65 kDa rPmpD monomer was almost completely degraded to smaller fragments ~22 kDa and ~35 kDa after 30 minutes. In contrast, oligomeric rPmpD showed a different proteolytic pattern, with a wider range of digest fragments and some undigested protein still detected, indicating a resistance to tryptic proteolysis. To determine the nature of biochemical interactions and address the mechanism of assembly of oligomeric rPmpD, we employed commonly used reducing agents in native and SDS-PAGE experiments.

### All cysteine residues can form disulphide bonds in monomeric rPmpD

**Fig 4:**
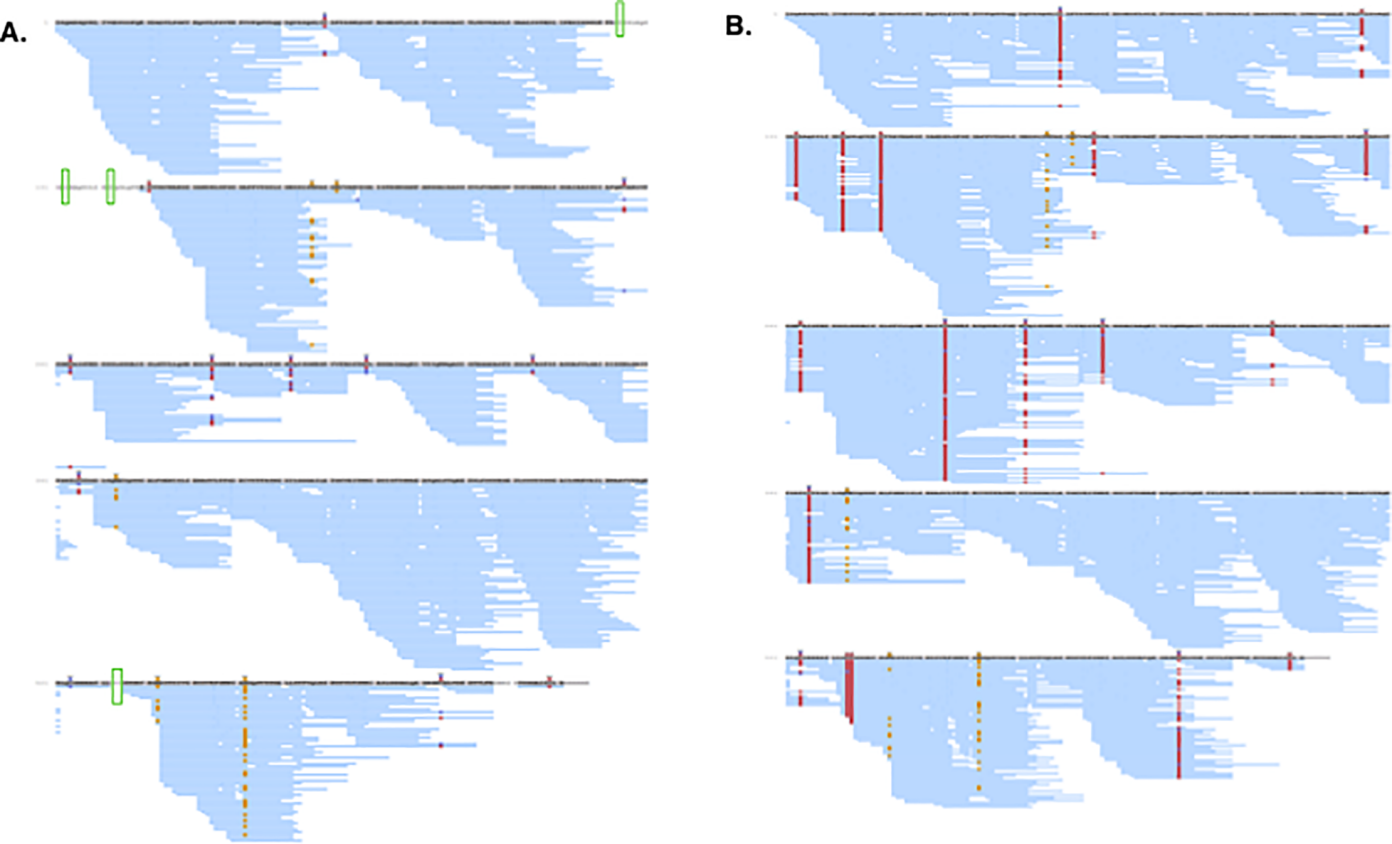
Differential alkylation of rPmpD and LC-MS/MS analysis. PEAKS identified peptides for (**A**) non-reduced and (**B**) reduced rPmpD are indicated by blue lines are mapped onto the amino acid sequence of His_6_-tagged rPmpD. Modified positions are annotated with coloured boxes and letters: **c** = carbamidomethylated cysteine (**blue**); **b** = methylthiolated cysteine (**red**); o = oxidised methionine (**yellow**). All cysteine-containing peptides show increased sampling of methylthiolated forms following reduction (**B**) suggesting that in the dominant proteoform(s) all 18 residues are likely disulfide-bonded. Methylthiolated residues at positions 124, 133, 143, 534 and 535 are identified by PEAKS exclusively post reduction (**B**), remaining unidentified in the non-reduced sample (**A**) (green boxes), suggesting that these cysteines are fastidiously disulphide-bonded, or remain non-solvent-exposed as peptides following digestion.

The aim of differential cysteine labeling was performed to identify the redox state of cysteines in the native state of the protein. High sequence coverage was obtained for both non-reduced (**Fig 4A)** and reduced **(Fig 4B**) rPmpD. Non-reduced monomeric rPmpD was found to contain multiple instances of cysteines in both carbamidomethylated and methylthiolated forms (**Fig 4A**), indicating that increased solvent-accessibility after enzymatic digestion allows for more complete alkylation of free thiols. Cysteine residues at positions 124, 133, 143, 534 and 535 were identified exclusively post reduction (**Fig 4B**), suggesting these positions are fastidiously disulfide bonded or remain solvent inaccessible even as post-enzymatic digested peptides. The remaining 13 cysteine residues were identified in both the reduced and non-reduced work-flows (**Fig 4A and 4B**). However, frequency of identification was much greater post reduction suggesting these 13 cysteine residues are predominantly oxidized in monomeric rPmpD. To gain a more precise quantitative comparison the data were analysed through Progenesis QI. Mascot derived peptide identifications were mapped onto LC-MS peak intensities and relative MS^1^ peak areas for carbamidomethylated and methylthiolated variants of the same peptide compared between non-reduced and reduced monomeric rPmpD, as shown in **Table 1**.

**Table 1:** Differential cysteine labeling of monomeric rPmpD.

Peptide, Charge, Alkylation Position in Peptide	Alkylation Position in Protein	Non-reduced—Carbamiodomethylated Relative Peak Area (%)	Non-reduced—Methylthiolated Relative Peak Area (%)	Reduced—Carbamiodomethylated Relative Peak Area (%)	Reduced—Methylthiolated Relative Peak Area (%)
DQVSSQGLICSFTSSNL, 2+, 10	60	0	2	0	99
DQVSSQGLICSFTSSNL, 3+, 10	60	0	1	0	99
DQVSSQGLICSFTSSNLDSPR, 2+, 10	60	0	1	0	100
DQVSSQGLICSFTSSNLDSPR, 3+, 10	60	0	0	82	0
QGVDQQDQVSSQGLICSF, 2+, 16	60	1	5	0	97
QGVDQQDQVSSQGLICSFTSSNL, 2+, 16	60	0	20	1	95
QGVDQQDQVSSQGLICSFTSSNL, 3+, 16	60	0	11	0	99
SVTNPVVFQGVDQQDQVSSQGLICSF, 2+, 24	60	0	7	0	98
SVTNPVVFQGVDQQDQVSSQGLICSF, 3+, 24	60	0	3	0	100
VEQSTLFSVTNPVVFQGVDQQDQVSSQGLICSFTSSNLDSPR, 4+, 31	60	0	2	0	98
ASCSSLEQGGACAAQSIL, 2+, 3, 12	133	0	0	0	100
DCQGLQVKHCTTAVNAEGSSAN, 2+, 2, 10	151	0	0	0	99
DCQGLQVKHCTTAVNAEGSSAN, 3+, 2, 10	151	0	1	0	99
HCTTAVNAEGSSANDHLGFGGGAFFVTGSLSGEK, 4+, 2	151	0	1	10	77
MPAGDMVVANCDGAISF, 2+, 11	197	0	3	0	98
MPAGDMVVANCDGAISFEGNSANF, 2+, 11	197	0	6	0	98
MPAGDMVVANCDGAISFEGNSANF, 3+, 11	197	0	4	0	96
SLYMPAGDMVVANCDGAISFEGNSANFANGGAIAASGK, 3+, 14	197	18	0	0	98
ALSGGAIAASSDIAFQNCAELVFK, 2+, 18	255	0	3	0	98
ALSGGAIAASSDIAFQNCAELVFK, 3+, 18	255	1	3	0	87
DIAFQNCAELVFKGNCAIGTE, 2+, 7, 16	255	0	1	0	100
DIAFQNCAELVFKGNCAIGTE, 3+, 7, 16	255	0	1	0	98
SGGAIAASSDIAFQNCAEL, 2+, 16	255	0	4	0	96
SGGAIAASSDIAFQNCAELVF, 2+, 16	255	0	3	0	97
KGNCAIGTEDKGSLGGGAISSL, 3+, 4	264	0	1	0	99
KGNCAIGTEDKGSLGGGAISSLGTVL, 3+, 4	264	0	2	0	98
DKGSLGGGAISSLGTVLLQGNHGITC, 2+, 26	295	0	1	0	99
DKGSLGGGAISSLGTVLLQGNHGITC, 3+, 26	295	0	2	0	98
GSLGGGAISSLGTVLLQGNHGITCDK, 2+, 24	295	0	1	0	99
GSLGGGAISSLGTVLLQGNHGITCDK, 3+, 24	295	0	2	0	98
GSLGGGAISSLGTVLLQGNHGITCDKNESASQGGAIFGK, 3+, 24	295	0	2	0	98
GSLGGGAISSLGTVLLQGNHGITCDKNESASQGGAIFGK, 4+, 24	295	0	2	0	98
GTVLLQGNHGITCDKNESASQGGAIF, 2+, 13	295	0	0	0	100
GTVLLQGNHGITCDKNESASQGGAIF, 3+, 13	295	0	2	0	98
LQGNHGITCDKNESASQGGAIF, 2+, 9	295	0	1	0	99
LQGNHGITCDKNESASQGGAIF, 3+, 9	295	0	3	0	96
DKNESASQGGAIFGKNCQIS, 2+, 17	312	0	0	0	100
DKNESASQGGAIFGKNCQIS, 3+, 17	312	0	1	0	99
DKNESASQGGAIFGKNCQISDNEGPVVFR, 3+, 17	312	0	0	0	100
DKNESASQGGAIFGKNCQISDNEGPVVFR, 4+, 17	312	0	1	0	99
GKNCQISDNEGPVVF, 2+, 4	312	0	1	0	99
NCQISDNEGPVVFR, 2+, 2	312	0	1	0	99
DSTACLGGGAIAAQEIVSIQNNQAGISFEGGK, 3+, 5	329	12	1	10	77
RDSTACLGGGAIAAQEIVSIQNNQAGISFEGGKASF, 3+, 6	329	0	2	0	98
RDSTACLGGGAIAAQEIVSIQNNQAGISFEGGKASF, 4+, 6	329	0	3	0	97
DISKNLGAISFSRTLCTTS, 2+, 16	396	0	0	0	100
DISKNLGAISFSRTLCTTS, 3+, 16	396	0	0	0	100
SRTLCTTSDLGQMEY, 2+, 5	396	0	1	0	98
EQNRLQCSEEEATL, 2+, 7	524	0	4	0	96
EQNRLQCSEEEATLL, 2+, 7	524	0	2	0	97
DFSRNIASLGGGALQASEGNCELV, 2+, 21	605	0	2	0	98
DFSRNIASLGGGALQASEGNCELV, 3+, 21	605	0	1	0	99
GGGALQASEGNCELVDNGY, 2+, 12	605	0	2	0	98
NIASLGGGALQASEGNCELVDNGYVLFR, 2+, 17	605	0	2	0	98
NIASLGGGALQASEGNCELVDNGYVLFR, 3+, 17	605	0	2	0	98
NIASLGGGALQASEGNCELVDNGYVLFRDNR, 3+, 17	605	0	2	0	98
GRVYGGAISCLR, 3+, 10	629	0	13	0	87
VYGGAISCLR, 1+, 8	629	0	0	0	100
VYGGAISCLR, 2+, 8	629	0	1	0	99
GGLEFASCSSLEQGGACAAQSILIHDCQGLQVK, 3+, 8, 17, 27	124, 133, 143	0	0	0	100
GGLEFASCSSLEQGGACAAQSILIHDCQGLQVK, 4+, 8, 17, 27	124, 133, 143	0	0	0	100
DLIFEKIKGGLEFASCSSLEQGGACAAQSILIH, 3+, 16, 25	124, 133	0	18	0	99
DLIFEKIKGGLEFASCSSLEQGGACAAQSILIH, 4+, 16, 25	124, 133	0	1	0	96
IHDCQGLQVKHCTTAVNAEGSSANDHLGF, 3+, 4, 12	143, 151	0	0	0	100
IHDCQGLQVKHCTTAVNAEGSSANDHLGF, 4+, 4, 12	143, 151	0	2	0	98
IHDCQGLQVKHCTTAVNAEGSSANDHLGF, 5+, 4, 12	143, 151	0	0	0	100
GNCAIGTEDKGSLGGGAISSLGTVLLQGNHGITCDK, 4+, 3, 34	264, 295	0	0	0	100
GNCAIGTEDKGSLGGGAISSLGTVLLQGNHGITCDKNESASQGGAIFGK, 4+, 3, 34	264, 295	0	2	0	98
GNCAIGTEDKGSLGGGAISSLGTVLLQGNHGITCDKNESASQGGAIFGK, 5+, 3, 34	264, 295	0	1	0	99
DKGSLGGGAISSLGTVLLQGNHGITCDKNESASQGGAIFGKNCQISDNEGPVVFR, 5+, 26, 43	295, 312	0	3	0	97
LQGNHGITCDKNESASQGGAIFGKNCQISDNEGPVVF, 3+, 9, 26	295, 312	0	0	0	100
LQGNHGITCDKNESASQGGAIFGKNCQISDNEGPVVF, 4+, 9, 26	295, 312	0	1	0	99
GKNCQISDNEGPVVFRDSTACL, 3+, 4, 12	312, 329	0	0	0	100
LQCSEEEATLLGCCGGGAVHGMDSTSIVGNSSVR, 3+, 3, 12, 14	524, 534, 535	0	0	0	100
GCCGGGAVHGMDSTSIVGNSSVRFGNNY, 3+, 2, 3	533, 534	0	0	0	100
GCCGGGAVHGMDSTSIVGNSSVRF, 3+, 2, 3	534, 535	0	0	0	100

Relative peak areas of carbamidomethylated and methylthiolated peptide forms pre- and post-reduction as extracted from Progenesis QI

Here, the measured peak areas for methylthiolated cysteine residues in the reduced work-flow are highly dominant. The peptide SLYMPAGDMVVANCDGAISFEGNSANFANGGAIAASGK containing Cys-197, is uniquely opposed to this trend, with its carbamidomethylated form measured to be most abundant, suggesting a higher rate of non-oxidized Cys-197. However, differential labelling for Cys-197 is also quantified from three further peptide precursors (MPAGDMVVANCDGAISFEGNSANF 2+ and 3+ and MPAGDMVVANCDGAISF 2+), and in these forms the post-reduction methylthiolated form accounts for over 90% of the ion intensity–indicating that the measurement in SLYMPAGDMVVANCDGAISFEGNSANFANGGAIAASGK may be a digestion artifact or a quantitative outlier. For all 18 cysteines the majority of sampled forms show greater than 95% relative peak area residing in the methylthiolated form post-reduction, suggesting that in monomeric rPmpD, all 18 cysteine residues are disuphide-bonded.

### Monomeric and oligomeric rPmpD mediate adherence to mammalian host cell lines

**Fig 5:**
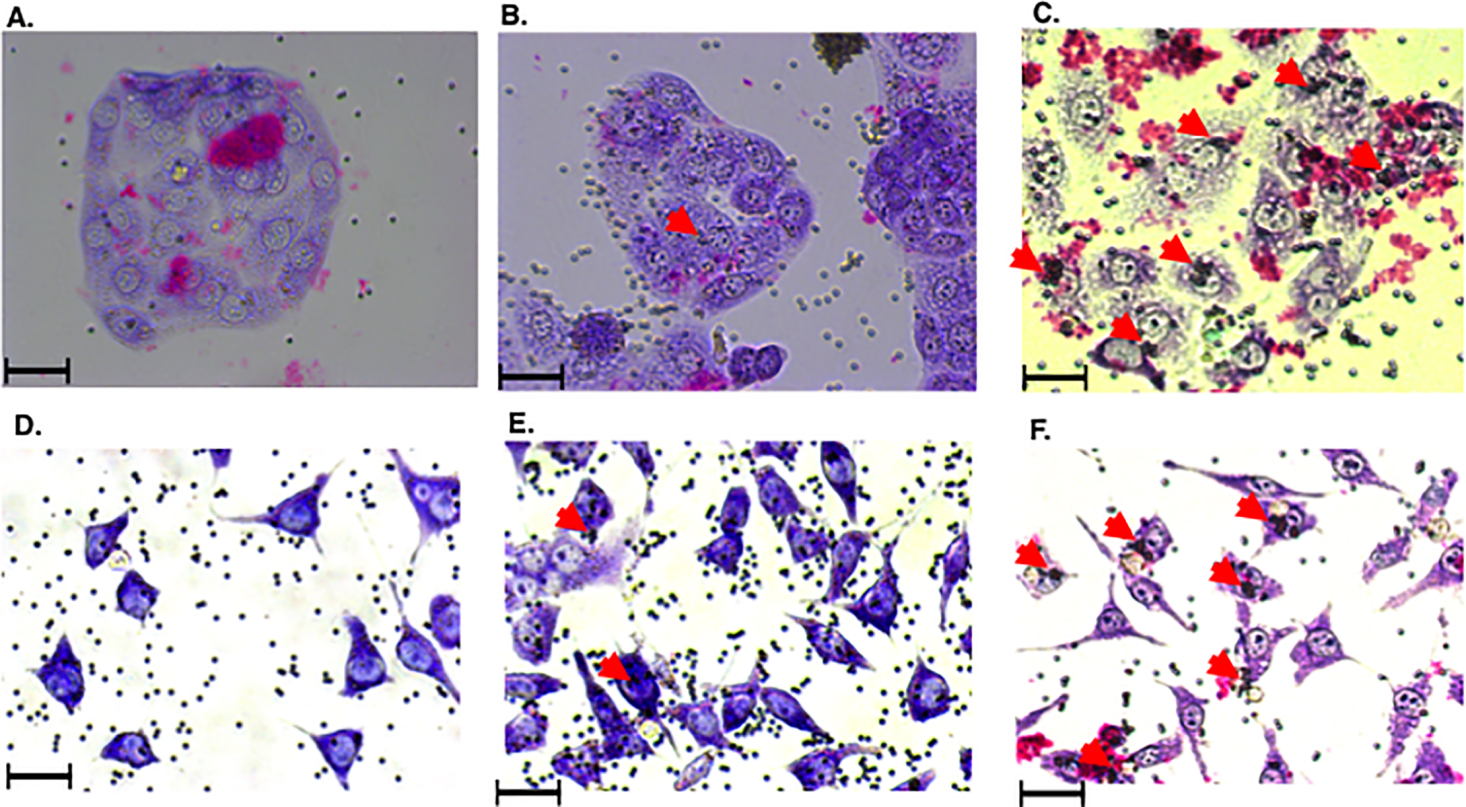
rPmpD-coated beads form adhesion clusters on the surface of mammalian cell lines. Syrian hamster kidney (Hak) cell lines (Panels **A-C**) or murine fibroblast (McCoy) cell lines (Panels **D-F**) were incubated with naked (**A,D**), BSA-coated (**B,E**) or rPmpD-coated (**C,F**) beads. Large clusters of rPmpD-coated beads are observed on the surfaces of both Hak and McCoy cell lines (red arrows). BSA-coated and naked beads do not demonstrate this aggregative property on the cell surface. Images were acquired at 10x magnification (Scale bar = 20 μm).

**Fig 6:**
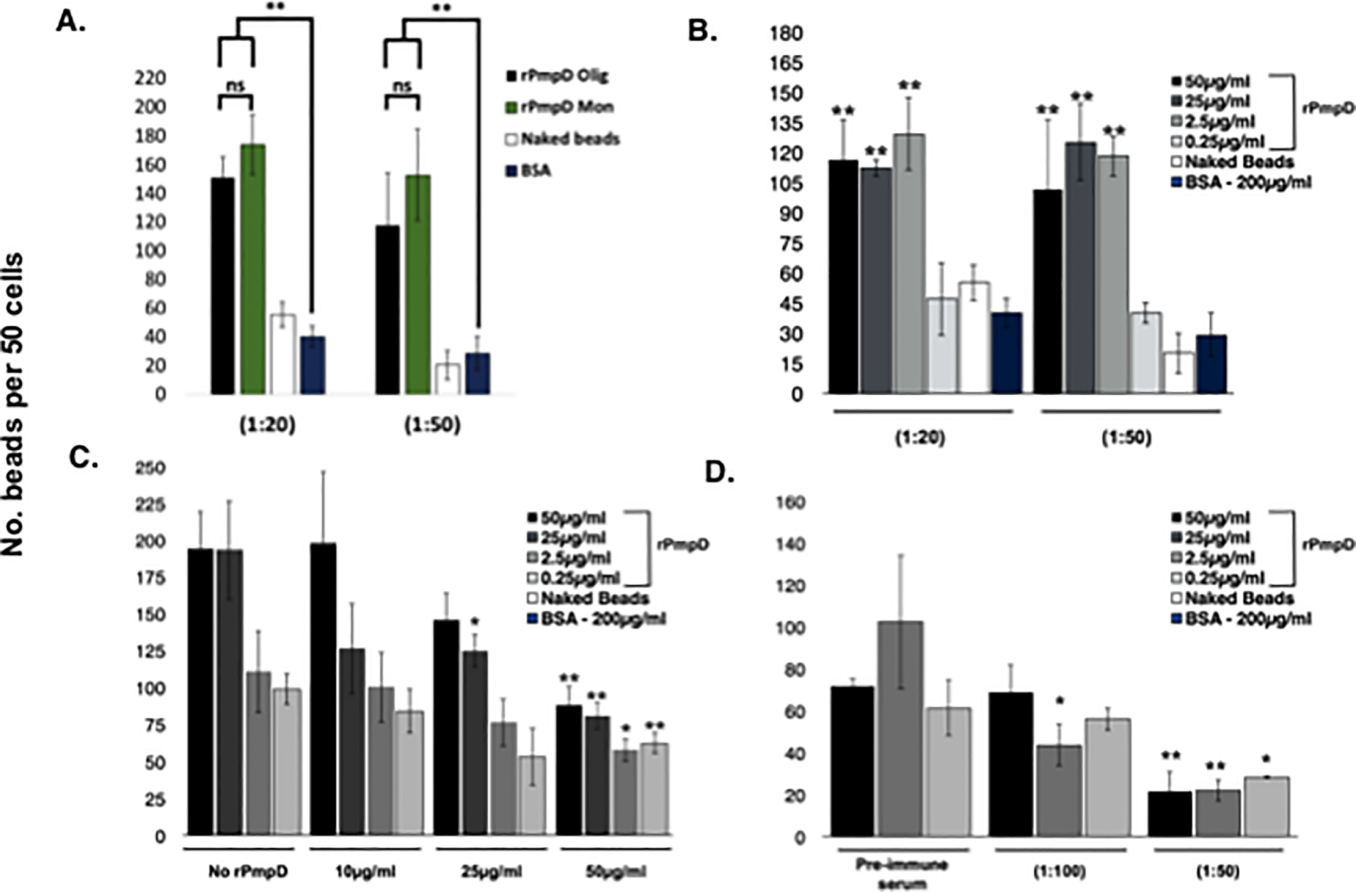
Adhesion of rPmpD-coated beads to Hak cells is inhibited by soluble rPmpD and anti-rPmpD serum. (**A**) Carboxylate-modified beads were coated with oligomeric or monomeric rPmpD (50 μg/ml) and compared to control (naked or 200 μg/ml BSA-coated beads) at two different bead dilutions. The number of rPmpD-coated beads adhered to 50 cells within triplicate fields of view was counted. Significantly enhanced adhesive capacity compared to naked or BSA-coated beads was observed, although no significant difference in adhesion was observed between beads coated with oligomeric and monomeric rPmpD proteoforms (**B**) Titration of oligomeric rPmpD (50 μg/ml-0.25 μg/ml) prior to bead coating shows a pronounced concentration-dependent reduction in adhesion. Significance values are reported relative to BSA-coated beads. (**C**) Soluble rPmpD competitor protein significantly reduces adhesion of rPmpD-coated beads in a dose-dependent manner, likely indicating competitive binding of putative host cell receptor(s). Significance is measured relative to pre-incubation with BSA only. **(D)** Heat-inactivated anti-rPmpD serum obtained from rPmpD-immunized mice abrogates adhesion of rPmpD-coated beads. Bead coating concentration of rPmpD varied from 50 μg/ml-2.5 μg/ml. Significance is measured relative to a 1:50 dilution of heat-inactivated pre-immune serum. All data are representative of 3 separate experiments, and presented as mean ± standard deviation. For all experiments, statistical significance was determined using a two-tailed t-test with GraphPad Prism 6. * = p≤0.05, ** p≤0.01, ***p≤0.001.

Adhesion assays incorporated 2μm diameter naked, rPmpD- or BSA-coated beads which were incubated with mammalian epithelial-like Hak cell or murine fibroblast McCoy cell lines as described in the Materials and Methods. Large clusters of rPmpD-coated beads can be seen attached to the cell surface in both cell types (red arrowheads- **Fig 5C and 5F**), in contrast to control beads (**Fig 5A, 5B, 5D and 5E**). These observations mimic the binding pattern of *Chlamydia pneumoniae* Pmp21-coated beads previously described by Molleken *et al*. [5], and demonstrate that *Ct* rPmpD also possesses adhesin-like properties. No significant differences were observed between the binding capacity of beads coated with monomeric or oligomeric rPmpD (Fig 6A). For subsequent competitive inhibition and neutralisation assays, beads were coated with oligomeric rPmpD. This is the dominant proteoform *in vitro*, and is also thought to be the more prevalent species on the oxidized membranes of infectious EBs *in vivo* [12].

Beads coated with 50 μg/mL of oligomeric or monomeric rPmpD showed a significant increase in adhesive capacity to Hak cell lines relative to naked and BSA-coated beads (**Fig 6A**). Titration of rPmpD bead coating concentrations from 50 μg/mL to 0.25 μg/mL was carried out, and two separate dilutions (1:20 and 1:50) of a 1% solution of polystyrene beads with Hak cells revealed a concentration-dependent reduction in bead binding (**Fig 6B**). A rPmpD bead coating concentration as low as 2.5 μg/mL still effected roughly four-fold greater adhesion (118±10) than beads coated with 200 μg/mL of BSA control protein (29±11), with no significant difference from higher 50 μg/mL or 25 μg/mL coating concentrations, indicating a saturating effect of protein concentration on the bead surface, or possible steric hindrance caused by higher concentrations of rPmpD that may affect binding.

Next, we sought to determine whether soluble rPmpD could block rPmpD-coated bead binding to Hak cells. Pre-incubation of Hak cells with soluble rPmpD (50 μg/mL-2.5 μg/mL) was carried out at 4°C, followed by washing and incubation with rPmpD-coated beads. No significant inhibition of rPmpD-coated bead attachment is seen following pre-treatment of cells with 10 μg/mL rPmpD. Increasing concentrations of soluble rPmpD competitor significantly abrogate bead binding to Hak cells relative to the negative control (pre-incubation with BSA) (**Fig 6C**). A 50 μg/mL rPmpD competitor concentration elicits ~55–60% reduction in bead adhesion.

Finally, to further investigate whether rPmpD was essential for adhesion, heat-inactivated pre-immune or anti-rPmpD serum from immunized C57BL/6 mice was pre-incubated with rPmpD-coated beads. We previously showed that this anti-rPmpD serum recognizes both rPmpD and UV-inactivated *Ct* EBs in ELISA assays, indicating recognition of native PmpD protein on chlamydial bacteria [8]. Two dilutions of immune serum (1:100 and 1:50) were incubated with rPmpD-coated beads (**Fig 6D**). Following addition of the serum-bead mixture to Hak cell monolayers, ~80% reduction in adhesion was observed with a 1:50 dilution of immune serum relative to the negative control (pre-immune serum).

## Discussion

*Ct* PmpD is a highly immunogenic chlamydial antigen following natural infection in humans with a range of serovars [26, 27]. In line with the degree and frequency of immunogenicity observed during the course of natural infections, we have recently shown that immunization with rPmpD elicits robust humoral and cell-mediated immunity that confer protection in a mouse model of genital *Ct* infection, and further confirmed the presence of ocular anti-rPmpD antibodies elicited following intramuscular immunization [8, 17]. Furthermore, other bacterial T5SSs have been included in licensed prophylactic acellular vaccines that include the *Bordetella pertussis* pertactin type Va autotransporter [28], and the more recently developed quadravalent 4CMenB vaccine (Bexsero), which contains the *Neisseria meningitidis* adhesin A (NadA) protein, a type Vc autotransporter [29]. In our study, we have utilized both biochemical and biophysical analytical techniques to further characterize rPmpD, a highly promising chlamydial vaccine antigen, importantly elucidating a previously undescribed mechanism of bacterial T5SS assembly involving an extensively disulphide-linked network of cysteine residues.

Early studies on the COMC showed that *Ct* EBs and RBs from both *Chlamydia psittaci* and *Ct* species were not solubilised by the anionic detergent SDS, unless reducing agents were present [21]. In addition, most of the chlamydial MOMP, that comprises roughly 65% of the COMC, was shown to exist in monomeric form within RBs, with membranes more readily dissociable using anionic detergents, while EBs possess a large proportion of disulphide-linked trimeric MOMP [24]. These findings were supported by our observations for rPmpD under non-reducing and reducing SDS PAGE, which indicate that even more extensive covalent disulphide bonding leads to formation of oligomeric complexes. Our data expand upon recent findings involving the 23 kDa *Chlamydia pneumoniae* Pmp21 passenger domain fragment, where a different mechanism of oligomeric interaction involving FxxN repeat motifs was proposed [18]. Indeed, we have observed that non-covalent interactions may also contribute to assembly of of rPmpD oligomers, evidenced by the dissociation of some monomeric protein in non-reducing SDS PAGE, although it is apparent that disulphide bonding is yet another important mechanism by which these cysteine-rich T5SS proteins interact, and it is likely that both types of interaction may occur in multiple chlamydial species.

The functional role for this extensively disulphide-bonded network is not well-characterised due to challenging experimental ramifications of the use of reducing and alkylating agents *in vivo*. However, it has previously been shown that the 67 kDa MOMP trimer was completely recalcitrant to digestion by trypsin even when incubated at 37°C for 20h, whereas monomeric MOMP was susceptible to cleavage by the enzyme [24]. Disulphide bonds were present between monomers in trimers and indeed, between trimers themselves, thus indicating that intermolecular disulphide interactions may be responsible for proteolytic recalcitrance. Proteolytic digests on monomeric and oligomeric rPmpD in our investigations show similar parallels to the respective MOMP proteoforms. Indeed, the array of polarized flower-like structures observed in the first electron micrograph images of PmpD on chlamydial EBs are consistent with the formation of oligomeric outer membrane structures [12].

LC-MS/MS analysis spans near complete sequence coverage of monomeric rPmpD, and, crucially, covers all cysteine residues. The presence of disulfide bonds was assessed by comparing the relative abundance of methylthiolated cysteine-containing peptides (pre- and post-reduction). Surprisingly, we found that widespread intramolecular cysteine-cysteine interactions contribute to the preservation of monomeric rPmpD secondary structure *in vitro*, though it is unclear in which form the protein predominantly exists *in vivo*, and whether this pattern of assembly remains, or is altered during each stage of biphasic development or secretion through the T5SS apparatus. All cysteine-containing peptides show increased sampling and intensity of methylthiolated forms post reduction suggesting that in monomeric rPmpD, all 18 residues exist in disulfide pairs. The LC-MS/MS data also suggest that there is a population of much lower abundance proteoforms in which a number of cysteines are present in the reduced form prior to chemical reduction. However, resolving these low abundance proteoforms is not possible from these data. For instance, there may be one proteoform where all cysteines are reduced or several forms in which two different cysteines are not cross-linked. This may suggest pleomorphic or misfolded conformational states of monomeric rPmpD subunits, which cannot be excluded due to the formidably extensive network of cysteine residues in the passenger domain.

Considering that rPmpD is likely comprised of variable homo-oligomeric species *in vitro*, and given the large numbers of cysteines dispersed throughout the protein, some residues may not have to be involved in the formation of higher-order oligomeric forms, forming intramolecular disulphide bonds, or existing as non-solvent-accessible free thiols. From the data presented here, these potentially include methylthiolated cysteines 124, 133, 143, 534 and 535 identified exclusively post-reduction by Peaks. Intramolecular disulphide bonds have also been shown to be important in the most well-characterized T5SS, the invasin protein from *Yersinia* sp., where the formation of the disulphide bond between Cys_906_ and Cys_982_ is critical for integrin binding [30]. Thus, cysteine interactions in T5SSs may fastidiously contribute to the preservation of optimal conformational structures, playing important roles in modulating the functionality of these proteins.

Here, we show that rPmpD monomers and oligomers play a role in mediating host cell adhesion, and in competitive inhibition assays, it is likely that binding of soluble rPmpD to a putative cellular receptor or extracellular matrix component in Hak cells competes for binding sites available to rPmpD-coated beads on the cell surface. In addition, anti-rPmpD serum abrogates bead binding to the cell surface in a dose-dependent manner. Hence, our data are consistent with previous findings in the field regarding the perceived function of Pmps as adhesins in other chlamydial species [5, 6, 18, 19]. It has been proposed that oligomerisation of bacterial cell surface proteins may be a means of enhancing adhesion through the incorporation of a greater concentration of binding sites [31]. Indeed, it has been shown for a truncated C-terminal 23 kDa passenger domain fragment of Pmp21, that oligomers mediate significantly greater adhesion to host cells than monomers [18]. However, in contrast, our data suggest no significant differences in adhesion between monomeric and oligomeric rPmpD proteoforms. This may be due to stark differences in the size of recombinant fragments used in the two studies. Luczak *et al*. utilise a 23 kDa C.pneumoniae Pmp21 domain, which contains only 2 FxxN repeat motifs [18]. In our study, we express the 65 kDa PmpD passenger domain fragment which is roughly three times the size, and a more likely physiological representation of an *in vivo* scenario, as it has previously been identified by mass spectrometry from infected cell lysates [13]. This rPmpD fragment contains 13 FxxN and 12 GGA(I,L,V) repeat motifs that are hypothesized to mediate adhesion in *C*.*pneumoniae* [19]. Hence, monomeric rPmpD may be sufficient to mediate adhesion as well as oligomeric proteoforms.

Certainly, the predominance of rPmpD in several full-length and processed forms on elementary and reticulate bodies (shown in electron micrographs), does suggest that the protein may exist in a variety of conformations on the chlamydial membrane *in vivo*, the functional relevance of which is not yet known [12]. Moreover, in addition to intermolecular interactions within the bacterial outer membrane itself, modification of thiol residues in cysteine-rich Pmps may initiate interaction with host cell components, facilitating internalization. Direct modification of thiols at the host cell interface by host-derived protein disulphide isomerase has been implicated in mediating chlamydial adhesion and invasion, as well as the internalization of important viral pathogens such as the human immunodeficiency virus and Newcastle disease virus, as well as cleavage of diphtheria toxin. Cysteine-rich Pmp adhesins could play roles in initiating invasion through similar mechanisms [32–35].

## Conclusion

In summary, the data gathered in this study provide advancement in our understanding of chlamydial polymorphic membrane protein secondary structure and intermolecular assembly *in vitro*. We have shown that in addition to non-covalent interactions, extensive disulphide-mediated covalent interactions play a role in formation of higher-order structures, which represent a previously undescribed mechanism of T5SS self-association that differs markedly from other bacterial species and may be unique to the *Chlamydiaceae*. Subsequent work aims to assess whether modification of these disulphide interactions influences protein function *in vitro* and *in vivo*, in order to gain a more comprehensive molecular understanding of chlamydial membrane protein assembly. PmpD also displays an adhesin-like function *in vitro*, and future studies will aim to identify putative host cell ligands, as knowledge of *Ct* binding to human epithelial cells still remains rudimentary.

## References

[1] Brunham RC, Rey-Ladino J. Immunology of Chlamydia infection: implications for a Chlamydia trachomatis vaccine. Nat Rev Immunol. 2005;5(2):149–61. Epub 2005/02/03. doi: 10.1038/nri1551 . 15688042 10.1038/nri1551

[2] Newman L, Rowley J, Vander Hoorn S, Wijesooriya NS, Unemo M, Low N, et al Global Estimates of the Prevalence and Incidence of Four Curable Sexually Transmitted Infections in 2012 Based on Systematic Review and Global Reporting. PLoS One. 2015;10(12):e0143304 Epub 2015/12/10. doi: 10.1371/journal.pone.0143304 ; PubMed Central PMCID: PMC4672879. 26646541 10.1371/journal.pone.0143304 PMC4672879

[3] Peipert JF. Clinical practice. Genital chlamydial infections. N Engl J Med. 2003;349(25):2424–30. Epub 2003/12/19. doi: 10.1056/NEJMcp030542 . 14681509 10.1056/NEJMcp030542

[4] Harris SR, Clarke IN, Seth-Smith HM, Solomon AW, Cutcliffe LT, Marsh P, et al Whole-genome analysis of diverse Chlamydia trachomatis strains identifies phylogenetic relationships masked by current clinical typing. Nat Genet. 2012;44(4):413–9, S1. doi: 10.1038/ng.2214 ; PubMed Central PMCID: PMCPMC3378690. 22406642 10.1038/ng.2214 PMC3378690

[5] Molleken K, Becker E, Hegemann JH. The Chlamydia pneumoniae invasin protein Pmp21 recruits the EGF receptor for host cell entry. PLoS Pathog. 2013;9(4):e1003325 Epub 2013/05/02. doi: 10.1371/journal.ppat.1003325 ; PubMed Central PMCID: PMC3635982. 23633955 10.1371/journal.ppat.1003325 PMC3635982

[6] Becker E, Hegemann JH. All subtypes of the Pmp adhesin family are implicated in chlamydial virulence and show species-specific function. Microbiologyopen. 2014;3(4):544–56. Epub 2014/07/06. doi: 10.1002/mbo3.186 ; PubMed Central PMCID: PMC4287181. 24985494 10.1002/mbo3.186 PMC4287181

[7] Yu H, Karunakaran KP, Jiang X, Brunham RC. Evaluation of a multisubunit recombinant polymorphic membrane protein and major outer membrane protein T cell vaccine against Chlamydia muridarum genital infection in three strains of mice. Vaccine. 2014;32(36):4672–80. Epub 2014/07/06. doi: 10.1016/j.vaccine.2014.06.002 ; PubMed Central PMCID: PMC4148050. 24992718 10.1016/j.vaccine.2014.06.002 PMC4148050

[8] Paes W, Brown N, Brzozowski AM, Coler R, Reed S, Carter D, et al Recombinant polymorphic membrane protein D in combination with a novel, second-generation lipid adjuvant protects against intra-vaginal Chlamydia trachomatis infection in mice. Vaccine. 2016;34(35):4123–31. Epub 2016/07/09. doi: 10.1016/j.vaccine.2016.06.081 ; PubMed Central PMCID: PMC4967447. 27389169 10.1016/j.vaccine.2016.06.081 PMC4967447

[9] Wells TJ, Tree JJ, Ulett GC, Schembri MA. Autotransporter proteins: novel targets at the bacterial cell surface. FEMS Microbiol Lett. 2007;274(2):163–72. Epub 2007/07/06. doi: 10.1111/j.1574-6968.2007.00833.x . 17610513 10.1111/j.1574-6968.2007.00833.x

[10] Grimwood J, Stephens RS. Computational analysis of the polymorphic membrane protein superfamily of Chlamydia trachomatis and Chlamydia pneumoniae. Microb Comp Genomics. 1999;4(3):187–201. Epub 1999/12/10. doi: 10.1089/omi.1.1999.4.187 . 10587946 10.1089/omi.1.1999.4.187

[11] Carlson JH, Porcella SF, McClarty G, Caldwell HD. Comparative genomic analysis of Chlamydia trachomatis oculotropic and genitotropic strains. Infect Immun. 2005;73(10):6407–18. doi: 10.1128/IAI.73.10.6407-6418.2005 ; PubMed Central PMCID: PMCPMC1230933. 16177312 10.1128/IAI.73.10.6407-6418.2005 PMC1230933

[12] Swanson KA, Taylor LD, Frank SD, Sturdevant GL, Fischer ER, Carlson JH, et al Chlamydia trachomatis polymorphic membrane protein D is an oligomeric autotransporter with a higher-order structure. Infect Immun. 2009;77(1):508–16. Epub 2008/11/13. doi: 10.1128/IAI.01173-08 ; PubMed Central PMCID: PMC2612253. 19001072 10.1128/IAI.01173-08 PMC2612253

[13] Kiselev AO, Skinner MC, Lampe MF. Analysis of pmpD expression and PmpD post-translational processing during the life cycle of Chlamydia trachomatis serovars A, D, and L2. PLoS One. 2009;4(4):e5191 Epub 2009/04/16. doi: 10.1371/journal.pone.0005191 ; PubMed Central PMCID: PMC2666266. 19367336 10.1371/journal.pone.0005191 PMC2666266

[14] Wehrl W, Brinkmann V, Jungblut PR, Meyer TF, Szczepek AJ. From the inside out—processing of the Chlamydial autotransporter PmpD and its role in bacterial adhesion and activation of human host cells. Mol Microbiol. 2004;51(2):319–34. Epub 2004/02/06. doi: 10.1046/j.1365-2958.2003.03838.x . 14756775 10.1046/j.1365-2958.2003.03838.x

[15] Wheelhouse NM, Sait M, Aitchison K, Livingstone M, Wright F, McLean K, et al Processing of Chlamydia abortus polymorphic membrane protein 18D during the chlamydial developmental cycle. PLoS One. 2012;7(11):e49190 Epub 2012/11/13. doi: 10.1371/journal.pone.0049190 ; PubMed Central PMCID: PMC3493501. 23145118 10.1371/journal.pone.0049190 PMC3493501

[16] Kari L, Southern TR, Downey CJ, Watkins HS, Randall LB, Taylor LD, et al Chlamydia trachomatis polymorphic membrane protein D is a virulence factor involved in early host-cell interactions. Infect Immun. 2014;82(7):2756–62. doi: 10.1128/IAI.01686-14 ; PubMed Central PMCID: PMCPMC4097629. 24733093 10.1128/IAI.01686-14 PMC4097629

[17] Badamchi-Zadeh A, McKay PF, Holland MJ, Paes W, Brzozowski A, Lacey C, et al Intramuscular Immunisation with Chlamydial Proteins Induces Chlamydia trachomatis Specific Ocular Antibodies. PLoS One. 2015;10(10):e0141209 Epub 2015/10/27. doi: 10.1371/journal.pone.0141209 ; PubMed Central PMCID: PMC4621052. 26501198 10.1371/journal.pone.0141209 PMC4621052

[18] Luczak SE, Smits SH, Decker C, Nagel-Steger L, Schmitt L, Hegemann JH. The Chlamydia pneumoniae Adhesin Pmp21 Forms Oligomers with Adhesive Properties. J Biol Chem. 2016;291(43):22806–18. Epub 2016/08/24. doi: 10.1074/jbc.M116.728915 ; PubMed Central PMCID: PMC5077213. 27551038 10.1074/jbc.M116.728915 PMC5077213

[19] Molleken K, Schmidt E, Hegemann JH. Members of the Pmp protein family of Chlamydia pneumoniae mediate adhesion to human cells via short repetitive peptide motifs. Mol Microbiol. 2010;78(4):1004–17. Epub 2010/11/11. doi: 10.1111/j.1365-2958.2010.07386.x ; PubMed Central PMCID: PMC2997323. 21062373 10.1111/j.1365-2958.2010.07386.x PMC2997323

[20] Crane DD, Carlson JH, Fischer ER, Bavoil P, Hsia RC, Tan C, et al Chlamydia trachomatis polymorphic membrane protein D is a species-common pan-neutralizing antigen. Proc Natl Acad Sci U S A. 2006;103(6):1894–9. Epub 2006/02/01. doi: 10.1073/pnas.0508983103 ; PubMed Central PMCID: PMC1413641. 16446444 10.1073/pnas.0508983103 PMC1413641

[21] Hatch TP, Allan I, Pearce JH. Structural and polypeptide differences between envelopes of infective and reproductive life cycle forms of Chlamydia spp. J Bacteriol. 1984;157(1):13–20. ; PubMed Central PMCID: PMCPMC215122. 6690419 10.1128/jb.157.1.13-20.1984 PMC215122

[22] Fan E, Chauhan N, Udatha DB, Leo JC, Linke D. Type V Secretion Systems in Bacteria. Microbiol Spectr. 2016;4(1). Epub 2016/03/22. doi: 10.1128/microbiolspec.VMBF-0009-2015 . 26999388 10.1128/microbiolspec.VMBF-0009-2015

[23] Liu S, Tobias R, McClure S, Styba G, Shi Q, Jackowski G. Removal of endotoxin from recombinant protein preparations. Clin Biochem. 1997;30(6):455–63. Epub 1997/08/01. . 9316739 10.1016/s0009-9120(97)00049-0

[24] Sun G, Pal S, Sarcon AK, Kim S, Sugawara E, Nikaido H, et al Structural and functional analyses of the major outer membrane protein of Chlamydia trachomatis. J Bacteriol. 2007;189(17):6222–35. doi: 10.1128/JB.00552-07 ; PubMed Central PMCID: PMCPMC1951919. 17601785 10.1128/JB.00552-07 PMC1951919

[25] Nishimura K, Tajima N, Yoon YH, Park SY, Tame JR. Autotransporter passenger proteins: virulence factors with common structural themes. J Mol Med (Berl). 2010;88(5):451–8. Epub 2010/03/11. doi: 10.1007/s00109-010-0600-y . 20217035 10.1007/s00109-010-0600-y

[26] Nunes A, Gomes JP, Mead S, Florindo C, Correia H, Borrego MJ, et al Comparative expression profiling of the Chlamydia trachomatis pmp gene family for clinical and reference strains. PLoS One. 2007;2(9):e878 Epub 2007/09/13. doi: 10.1371/journal.pone.0000878 ; PubMed Central PMCID: PMC1963315. 17849007 10.1371/journal.pone.0000878 PMC1963315

[27] Tan C, Hsia RC, Shou H, Haggerty CL, Ness RB, Gaydos CA, et al Chlamydia trachomatis-infected patients display variable antibody profiles against the nine-member polymorphic membrane protein family. Infect Immun. 2009;77(8):3218–26. Epub 2009/06/03. doi: 10.1128/IAI.01566-08 ; PubMed Central PMCID: PMC2715660. 19487469 10.1128/IAI.01566-08 PMC2715660

[28] Cherry JD, Gornbein J, Heininger U, Stehr K. A search for serologic correlates of immunity to Bordetella pertussis cough illnesses. Vaccine. 1998;16(20):1901–6. Epub 1998/10/31. . 9796041 10.1016/s0264-410x(98)00226-6

[29] Serruto D, Bottomley MJ, Ram S, Giuliani MM, Rappuoli R. The new multicomponent vaccine against meningococcal serogroup B, 4CMenB: immunological, functional and structural characterization of the antigens. Vaccine. 2012;30 Suppl 2:B87–97. Epub 2012/05/25. doi: 10.1016/j.vaccine.2012.01.033 ; PubMed Central PMCID: PMC3360877. 22607904 10.1016/j.vaccine.2012.01.033 PMC3360877

[30] Hamburger ZA, Brown MS, Isberg RR, Bjorkman PJ. Crystal structure of invasin: a bacterial integrin-binding protein. Science. 1999;286(5438):291–5. Epub 1999/10/09. . 10514372 10.1126/science.286.5438.291

[31] Ling H, Boodhoo A, Hazes B, Cummings MD, Armstrong GD, Brunton JL, et al Structure of the shiga-like toxin I B-pentamer complexed with an analogue of its receptor Gb3. Biochemistry. 1998;37(7):1777–88. Epub 1998/03/04. doi: 10.1021/bi971806n . 9485303 10.1021/bi971806n

[32] Ryser HJ, Mandel R, Ghani F. Cell surface sulfhydryls are required for the cytotoxicity of diphtheria toxin but not of ricin in Chinese hamster ovary cells. J Biol Chem. 1991;266(28):18439–42. Epub 1991/10/05. . 1655751

[33] Jain S, McGinnes LW, Morrison TG. Thiol/disulfide exchange is required for membrane fusion directed by the Newcastle disease virus fusion protein. J Virol. 2007;81(5):2328–39. Epub 2006/12/08. doi: 10.1128/JVI.01940-06 ; PubMed Central PMCID: PMC1865930. 17151113 10.1128/JVI.01940-06 PMC1865930

[34] Ou W, Silver J. Role of protein disulfide isomerase and other thiol-reactive proteins in HIV-1 envelope protein-mediated fusion. Virology. 2006;350(2):406–17. Epub 2006/03/02. doi: 10.1016/j.virol.2006.01.041 . 16507315 10.1016/j.virol.2006.01.041

[35] Davis CH, Raulston JE, Wyrick PB. Protein disulfide isomerase, a component of the estrogen receptor complex, is associated with Chlamydia trachomatis serovar E attached to human endometrial epithelial cells. Infect Immun. 2002;70(7):3413–8. doi: 10.1128/IAI.70.7.3413-3418.2002 ; PubMed Central PMCID: PMCPMC128041. 12065480 10.1128/IAI.70.7.3413-3418.2002 PMC128041

